# Oqtans: a Galaxy-integrated workflow for quantitative transcriptome analysis from NGS Data

**DOI:** 10.1186/1471-2105-12-S11-A7

**Published:** 2011-11-21

**Authors:** Sebastian J Schultheiss, Géraldine Jean, Jonas Behr, Regina Bohnert, Philipp Drewe, Nico Görnitz, André Kahles, Pramod Mudrakarta, Vipin T Sreedharan, Georg Zeller, Gunnar Rätsch

**Affiliations:** 1 Machine Learning in Biology Group, Friedrich Miescher Laboratory of the Max Planck Society, 72076 Tübingen, Germany; 2 Department of Software Engineering and Theoretical Computer Science, Technical University Berlin, 10578 Berlin, Germany; 3 Structural and Computational Biology Unit, European Molecular Biology Laboratory, 69117 Heidelberg, Germany

## Background

The current revolution in sequencing technologies allows us to obtain a much more detailed picture of transcriptomes via RNA-Sequencing. We have developed the first integrative online platform, oqtans, for quantitatively analyzing RNA-Seq experiments. Our approach of providing a self-contained machine image with the accessible, transparent Galaxy framework [1] minimizes the risk of using a third-party web service for data analysis. These services often disappear a few years after publication and render results irreproducible [2]. With oqtans, bioinformatics becomes reproducible by providing analysis building blocks for a customized workflow of read mapping, transcript reconstruction and quantitation as well as differential expression analysis.

## Method

Oqtans includes a comprehensive machine-learning-powered toolsuite developed by the authors for NGS data analysis. PALMapper is a short-read mapper which efficiently computes both unspliced and spliced alignments at high accuracy by taking advantage of base quality information and computational splice site predictions [3]. mTIM is a transcript reconstruction method, which exploits features derived from RNA-seq read alignments and from computational splice site predictions to infer the exon-intron structure of the corresponding transcripts. rQuant is based on quadratic programming. It simultaneously estimates biases inherent in library preparation, sequencing, and read mapping, and accurately determines the abundances of given transcripts [4]. rDiff is a set of statistical test techniques that determine significant differences between two RNA-seq experiments to find differentially expressed regions with or without knowledge of transcripts.

## Results

**Figure 1:**
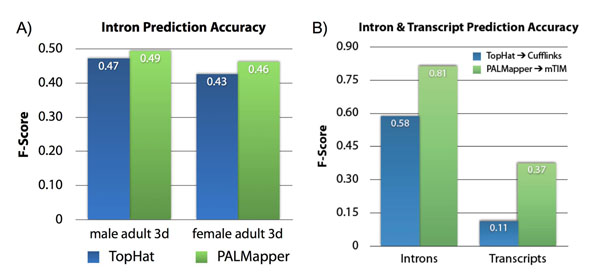
A) Accuracy (F-score) of intron predictions in 3-day-old adults of D. melanogaster with aligners PALMapper (green) and TopHat (blue). B) Accuracy of intron predictions with the same aligners and transcript predictions with mTIM (green) and Cufflinks (blue) on C. elegans RNA-seq transcriptome data.

We compare predictions to the published annotation at the intron and transcript levels. The performance of read aligners is shown in Fig. 1A from D. melanogaster data, and transcript segmentation tools in Fig. 1B, on C. elegans. Our tools, PALMapper and mTIM, outperform TopHat [5] and Cufflinks [6]. Oqtans is available free and open-source, from http://oqtans.org as a virtual machine for cloud computing environments, and ready to use on our public compute cluster at http://bioweb.me/mlb-galaxy.
